# Cohort Profile Update: The National Prostate Cancer Register of Sweden and PCBase

**DOI:** 10.1093/ije/dyaf172

**Published:** 2025-10-14

**Authors:** Marcus Westerberg, Lennart Holm, Hans Garmo, Pär Stattin, Rolf Gedeborg

**Affiliations:** Department of Surgical Sciences, Uppsala University, Uppsala, Sweden; Department of Surgical Sciences, Uppsala University, Uppsala, Sweden; Department of Surgical Sciences, Uppsala University, Uppsala, Sweden; Department of Surgical Sciences, Uppsala University, Uppsala, Sweden; Department of Surgical Sciences, Uppsala University, Uppsala, Sweden

**Keywords:** prostate cancer, prostate-specific antigen test, prostate biopsy, magnetic resonance imaging, chemotherapy

Key Features

- The first version of the Prostate Cancer data Base Sweden (PCBase) was created in 2009 by linking the National Prostate Cancer Register of Sweden with nationwide population-based healthcare registers and demographic databases to provide a database for research on prostate cancer.
- Longitudinal data with results from prostate-specific antigen testing, prostate biopsies, magnetic resonance imaging of the prostate, and the use of chemotherapy are critical for studies on prostate cancer screening and progression.
- Such individual-level information is not available in any Swedish healthcare register but has now been collected from healthcare IT systems in all 21 regions in Sweden via the new PCBase with Extended Treatment and Endpoint Data.
- Data were collected on all men in Sweden aged >30 years, irrespective of prostate cancer diagnosis, between 1998 and 2024 (>5 million men).
- For proposals for collaborations, contact par.stattin@uu.se.

## The original cohort

The National Prostate Cancer Register (NPCR) of Sweden is a clinical cancer register with the aim of increasing the quality of care for men with prostate cancer by providing data to assess adherence to national guidelines [1, 2]. Since 1998, the NPCR has captured data on >98% of all men diagnosed with prostate cancer and registered in the Swedish Cancer Register, to which reporting is mandated by law [3–5].

The NPCR contains comprehensive data on cancer characteristics at date of diagnosis, diagnostic workup including prostate biopsies and imaging, and primary treatment strategy. Patient reported outcomes (PROM) forms are distributed before and 1 year after radical treatment.

In the Prostate Cancer data Base Sweden (PCBase), matched prostate cancer-free control men have been added. Further data on both prostate cancer cases and the control men have been linked from other nationwide population-based healthcare registers and demographic databases by using unique Swedish personal identity numbers. These additional data sources include the Patient Register, the Prescribed Drug Register, the Cause of Death Register, the Register of the Total population, the Cancer Register, the Multi-Generation Register, the Longitudinal database on socioeconomic factors (LISA), and the National Diabetes Register, as detailed in previous cohort profiles [3, 4].

PCBase has been updated every third year to allow longer follow-up and the inclusion of new incident cases and matched control men. Since its inception in 2009, PCBase has been the basis for 250 peer-reviewed articles on a wide range of topics [6, 7].

## What is the reason for the new data collection?

### Prostate cancer screening

Screening using prostate-specific antigen (PSA) testing and early radical treatment has been shown to reduce prostate cancer mortality in randomized clinical trials [8, 9]. The optimal screening protocol in terms of test frequency, mode of investigation, and age limits remains to be determined. It is, however, very challenging to study the impact of different screening parameters. Due to the very long disease trajectory for prostate cancer, it will take up to 40 years of follow-up before all downstream effects of screening and early prostate cancer treatment can be observed in a population.

One strategy in this context is to utilize the large variation in opportunistic screening intensity between regions and over time in Sweden as a natural experiment that enables studies on how to optimize screening. However, longitudinal individual-level data on key components in prostate cancer screening, i.e. PSA testing, prostate biopsies, and magnetic resonance imaging (MRI) of the prostate, are not registered in any national healthcare register in Sweden. We therefore collected these data directly from electronic health records and laboratory information systems at regional or hospital levels and from private healthcare providers.

### Active surveillance

Most men with favourable-risk prostate cancer are followed through active surveillance, which currently consists of regular PSA testing, prostate MRIs, and, if there are signs of progression, prostate biopsies [2]. Around half of the men on active surveillance will eventually transition to radical treatment due to perceived progression [10, 11], although there is a lack of population-based data on the proportion of men who transit from active surveillance to radical treatment.

### Progression after radical treatment

The disease trajectory for men with prostate cancer is often decades long and serum PSA is a critical component during follow-up. Until now, PSA during follow-up has not been available in PCBase. With longitudinal data on PSA, MRIs, and biopsies in men with prostate cancer, we can assess the risk of cancer relapse after radical prostatectomy and radical radiotherapy. For men on active surveillance, we will be able to study triggers for transition to radical treatment on prognosis for downstream events.

### Treatments for advanced prostate cancer

Chemotherapy is used as a primary treatment in men with *de novo* metastatic prostate cancer and in men with castration-resistant prostate cancer (CRPC) signalled by rising PSA while on gonadotropin-releasing hormone (GnRH) agonists [2]. For example, until now, it has not been possible to define when the CRPC occurred and assess whether these men received chemotherapy. We have now collected individual-level data on PSA and the use of chemotherapy from regional information technology (IT) systems.

Data on filled prescriptions for androgen deprivation therapy (ADT), either oral with bicalutamide or injections with GnRH agonists and antagonists, are available in the Prescribed Drug Register with the exception of five regions in Sweden in which GnRHs are distributed via outpatient clinics. Therefore, we have collected individual-level data on administered GnRH from regional IT systems.

## What will be the new areas of research?

The most recent version of the original cohort included men diagnosed with prostate cancer until 2020, matched control men without prostate cancer and relatives of the men with prostate cancer, and data from national registers. PCBase with Extended Treatment and Endpoint Data (Xtend) will open up entirely new areas for population-based research, providing comprehensive longitudinal data from both national registers and regional healthcare databases with high granularity and inclusion until 2024, not only on all men diagnosed with prostate cancer, but also on all prostate-cancer-free men.

### Screening

Data on prostate-cancer-free men will be used to model the relationship between screening intensity, prostate cancer diagnosis, and subsequent outcomes at an individual level. This group consists of PSA-naïve men who have a PSA history but are biopsy-naïve, and men with both a PSA history and negative biopsies. By use of the large number of repeated PSA tests, we can predict PSA trajectories based on the observed velocities (i.e. rate of change over time) and the likelihood of a negative biopsy versus a prostate cancer diagnosis, taking the full history of PSA tests and biopsies into account. The prediction approach will enable us to compare different prostate cancer screening protocols by tweaking simulated protocols regarding modifiable parameters such as time between testing, cutoffs for workup, age limits, and the effect of prostate MRIs before a decision on prostate biopsy is made.

These models can also incorporate the influence of demographic and socioeconomic factors, comorbidity, and family history. Due to the long disease course of prostate cancer, such simulation models are an essential complement to standard study designs to inform and support public health decisions on screening.

### The disease trajectory

In men diagnosed with prostate cancer, we will be able to follow the entire prostate cancer disease trajectory in terms of progression. The complete disease trajectory for men on active surveillance includes triggers for transition from active surveillance to radical treatment; PSA tests, MRIs, and prostate biopsies during follow-up can now be assessed in an entire population. The risk of PSA relapse after radical prostatectomy and radical radiotherapy can also be studied in a large population-based cohort, including the prognostic implications and effects of subsequent treatment.

With the addition of data on GnRH administrations from healthcare records to the information previously retrieved from the Prescribed Drug Register, we now have complete data on the medical treatment of men with prostate cancer. When combined with data on PSA, this will allow us to assess the incidence of CRPC, treatment patterns, and additional treatment due to PSA progression in men on ADT, chemotherapy, and other systemic treatments for advanced prostate cancer.

## Who is in the cohort?

**Figure 1.**
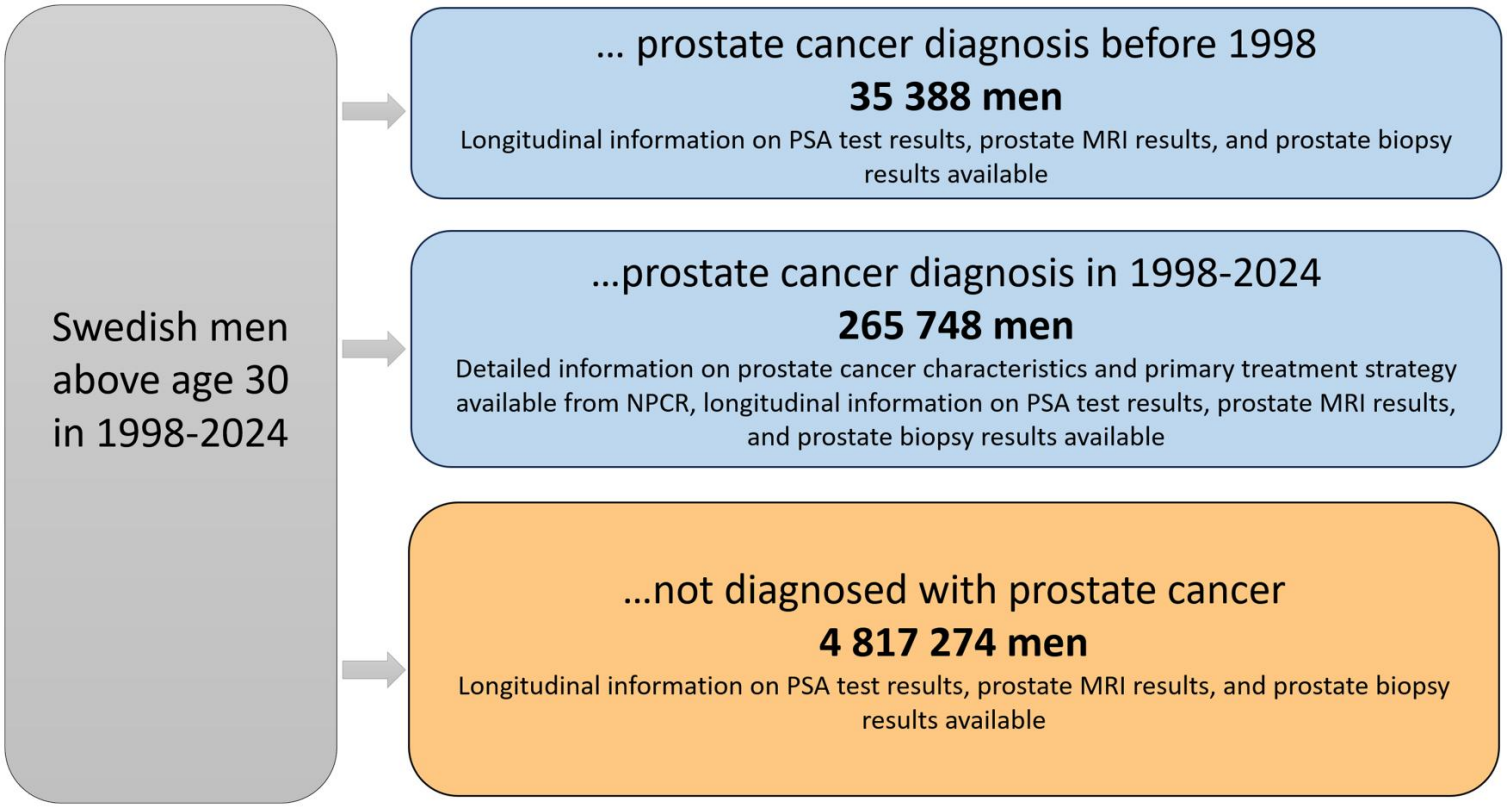
Men with and without prostate cancer included in PCBase Xtend. Prostate cancer cases were identified in the Cancer Register and the NPCR. Relatives were identified in the Multi-Generation Register.

All men with permanent residence in Sweden identified via a unique Swedish person identity number above the age 30 years are included in PCBase Xtend, including 35 388 men with prostate cancer diagnosed before 1998, 265 748 men diagnosed with prostate cancer between 1998 and 2024, and 4 817 274 men not diagnosed with prostate cancer (Fig. 1). In addition, all male siblings, sons, and fathers above the age of 30 years, also including those who died before 1998, are available via the Multi-Generation Register.

## What has been measured?

**Figure 2.**
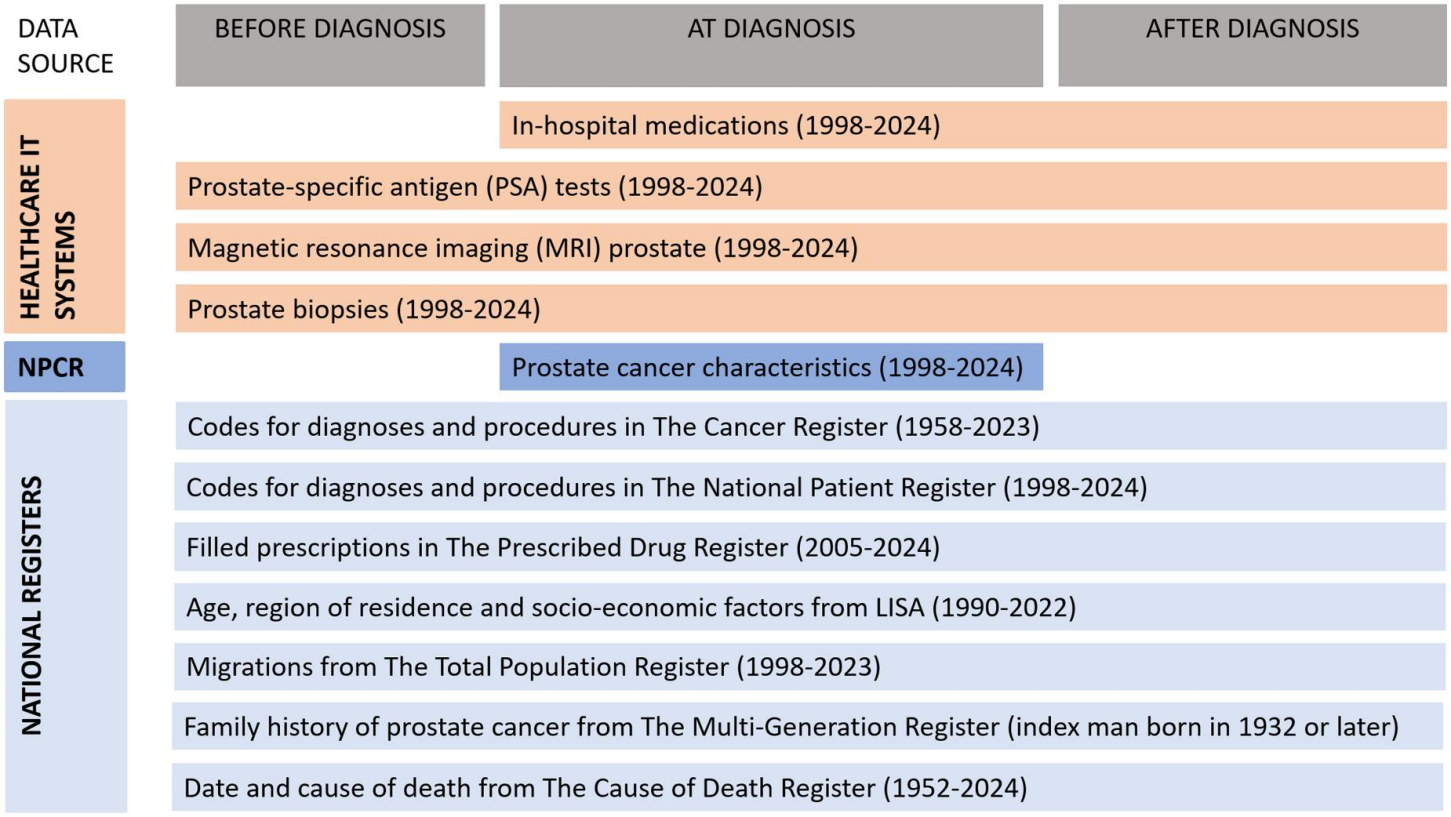
Data sources and types of longitudinal data in PCBase Xtend. Data in some national registers are currently only available until 2022–2023, but future updates of PCBase Xtend will extend the availability of data to 2024 and beyond.

To address the lack of critical data on PSA testing, prostate biopsies, prostate MRIs, and relevant medications, we initiated a nationwide effort to collect selected healthcare data from all 21 regions in Sweden. Following ethical approval, we requested individual-level data from regional healthcare IT systems, encompassing both public and private providers, including primary care. Data sources included electronic healthcare records, medication modules, chemotherapy systems, laboratory information systems, and radiology information systems (Fig. 2). Specifically, we collected:

- PSA tests: date for testing and levels of PSA;
- prostate biopsies: date and full histopathological report, including Gleason scores;
- prostate MRIs: date and narrative reports, including Prostate Imaging-Reporting and Data, System assessment, extracapsular extension, seminal vesicle involvement, and prostate volume;
- medications administered by healthcare staff: ATC codes L01**** (antineoplastic agents including chemotherapy), L02AE** (gonadotropin-releasing hormone analogues), L02BB** (anti-androgens), or L02BX** (other hormone antagonists), including date of administration, active substance, and administered dose.

Data requests were submitted sequentially to public healthcare regions and private providers. In some cases, separate applications were required for individual hospitals. To streamline the process, we developed a structured application package detailing the data specification, legal and ethical considerations, and data transfer and protection protocols. Administrative procedures varied widely across regions, with notable differences in the readiness to handle such requests. Online meetings and repeated personal contact were essential in navigating this complex and often time-consuming process. The average time from application to data delivery was 13 months (range 2–40 months). Early quality-assurance checks (see below) were crucial and identified several instances of incorrect data extractions, which were subsequently corrected.

These data were collected for all individuals in the updated cohort. In addition, information from national healthcare registers and demographic databases previously used in PCBase has now also been extracted for all men in the updated cohort, including the 4 817 274 men without a prostate cancer diagnosis (Fig. 2).

**Figure 3.**
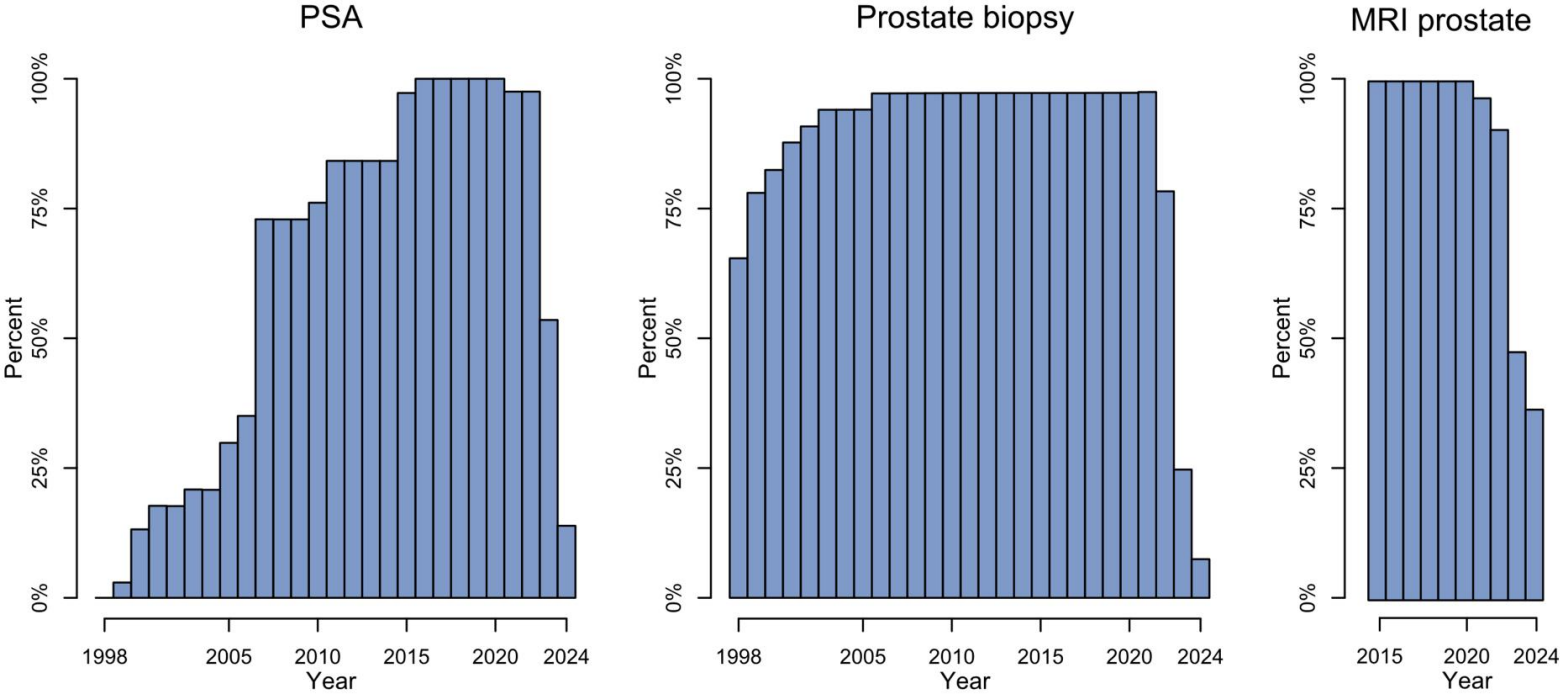
Annual estimated coverage compared with all Swedish men aged >30 years. Men were included if they were alive and resided in Sweden with a known region of residence on 1 January in the corresponding year and contributed to the nominator if they resided in a region in which the corresponding data type was considered available. MRI was considered to have been covered from 2015 at the earliest, although the database also contains MRIs performed before 2015.

The national annual coverage of PSA tests, prostate biopsies, and prostate MRIs—defined as the proportion of men residing in a region in which corresponding data were captured in PCBase Xtend—varied over time (Fig. 3). Between 2016 and 2020, the coverage was complete (100%) for PSA and MRI data, and 97% for biopsy data. The coverage of ADT was complete from July 2005 onward, while chemotherapy data availability varied substantially across regions. Future updates will continue to improve coverage.

## What has it found? Key findings and publications

### Research output from PCBase

PCBase has been the basis for 250 peer-reviewed articles since its inception in 2009 and areas that have been investigated include oncological outcomes, pattern of care, drug utilization studies, rare adverse drug effects, and comorbidity [6, 7]. Some examples of studies based on the most recent version of PCBase are:

- Accurate assessment of life expectancy is crucial in men with localized prostate cancer. We created a drug comorbidity index based on entries in the Prescribed Drug Register [12–14] and a multidimensional diagnosis-based comorbidity index based on International Classification of Diseases (ICD) codes in the Patient Register [15]. Both of these indices outperformed the commonly used Charlson Comorbidity Index and a prostate cancer comorbidity index [12, 15, 16]. Patient age and these two new indices provided accurate predictions of life expectancy at a group level [17], which are useful in observational studies of adherence to guidelines and for descriptive and prognostic purposes [18–22].
- In a target trial emulation against a randomized clinical trial (RCT) on prostatectomy vs radiotherapy, adjustment for comorbidity by the use of these two new indices resulted in a similar risk of all-cause death to that seen in the RCT [23]. Similar results were also obtained in a broader study group, showing that these indices can be used to reduce confounding bias in observational studies on treatment effectiveness.
- In two studies using simulations of long-term outcomes in men without active treatment, our results indicated that active surveillance is safe for men aged >65 years who are diagnosed with low-risk prostate cancer and that watchful waiting is appropriate for men with a life expectancy of <10 years [24, 25].
- We compared functional outcomes after radical prostatectomy shortly after diagnosis with those after surgery performed after a period of active surveillance [26]. The results indicated little detrimental effect of a period of active surveillance before surgery.

### Pilot studies in PCBase Xtend

In an early pilot study, we evaluated the effects of pre-biopsy MRI [27]. After MRI was introduced for diagnostic workup, there was a decrease in biopsy frequency, a smaller proportion of Gleason score 6, and a larger proportion of Gleason score 7, supporting that the use of MRI reduces the number of unnecessary biopsies.

In another early pilot, we studied triggers for the transition from active surveillance to radical treatment in men diagnosed in 2008–2020. The proportion of men who had progression of their cancer grade as a trigger has increased over time, indicating an increased quality of care [28].

In yet another pilot study following the collection of data from the first 12 regions, we assessed the risk of PSA relapse after radical prostatectomy [21]. The 10-year risk of PSA relapse was 34%, with a wide range in the 10-year risk of prostate cancer death after relapse, ranging from 12% in men with PSA persistence to 2% for men with low-risk relapse.

### Annual number of PSA-tested men

**Figure 4.**
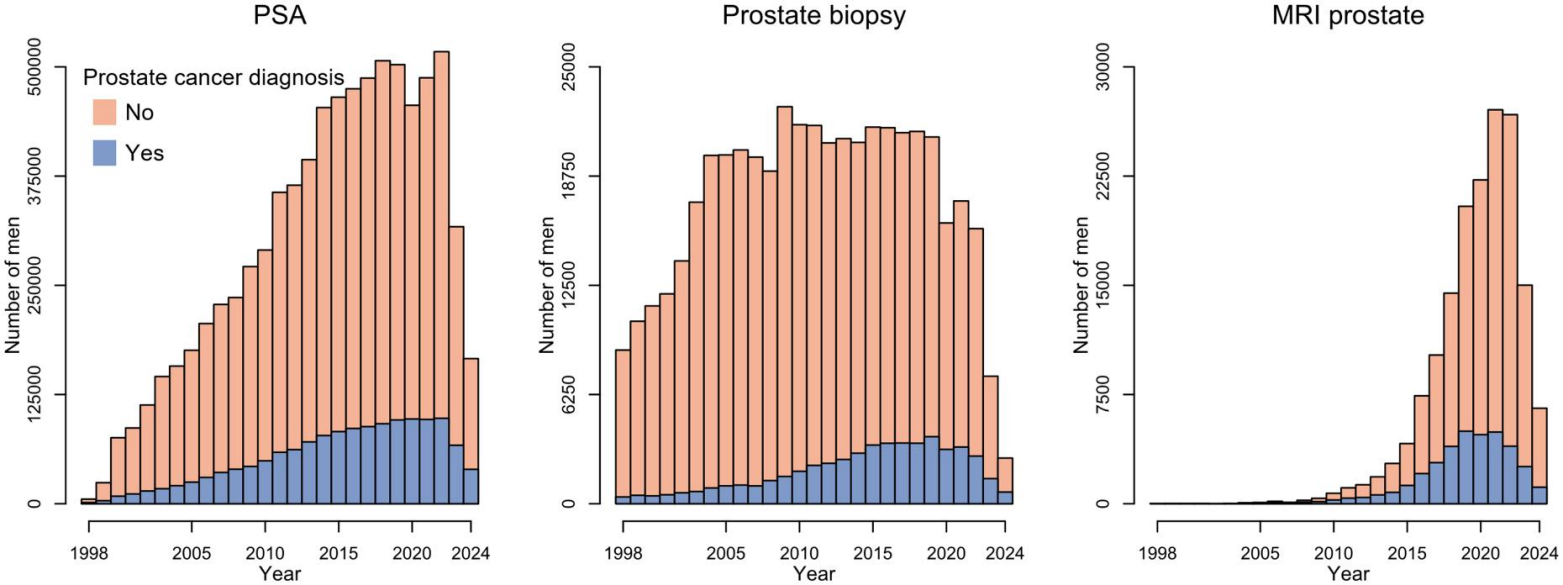
Annual number of men who underwent PSA testing, prostate biopsy, and prostate MRI. Prostate cancer cases were identified in the Cancer Register and the NPCR, and men were considered to have prostate cancer if diagnosed before 1 January in the corresponding year.

The annual number of PSA-tested men generally increased over time and there were up to ∼500 000 men per year who were PSA-tested in 2016–2020, when the coverage was complete (Fig. 4). Around 21 000 men per year underwent a biopsy of the prostate during the same period and the number of men who underwent an MRI increased from 7400 in 2016 to 22 000 in 2020.

## What are the main strengths and weaknesses?

PCBase Xtend contains comprehensive population-based data on prostate cancer, not only covering the disease and treatment trajectory, but also characterizing the period before diagnosis. The nationwide population-based cohort with virtually complete longitudinal data on PSA, prostate MRIs, and prostate biopsies will be the basis for studies on a wide range of topics. Longitudinal data from healthcare IT systems on critical variables have been added to the already comprehensive database obtained by using linkages to national healthcare registers.

Collecting data directly from healthcare IT systems and curating these data to a research-standard database proved to be demanding but feasible. The covered time periods differed between regions due to different calendar times for the implementation of new IT systems and the extent to which old data had been transferred to new IT systems. The duration of follow-up in some national registers, including the Cancer Register and the Cause of Death register, is currently limited to 2022–2024, but future updates will mitigate this limitation (Fig. 2).

## Can I get hold of the data? Where can I find out more?

There have been a large number of collaborations based on PCBase since its inception and there are several ongoing collaborations. The PCBase reference group welcomes proposals for collaboration; for further information, please contact par.stattin@uu.se (see ‘Data availability’ statement for details).

## Ethics approval

PCBase Xtend has been approved by the Swedish Research Ethics authority (Dnr 2022–05083-02) as well as the National Board of Health and Welfare and Statistics Sweden.

## Data Availability

Data used in the present study were extracted from PCBase, which is based on the NPCR of Sweden and linkage to several national health data registers. The data cannot be shared publicly because the individual-level data contain potentially identifying and sensitive patient information, and cannot be published due to legislation and ethical approval (https://etikprovningsmyndigheten.se). Use of the data from national health data registers is further restricted by the Swedish Board of Health and Welfare (https://www.socialstyrelsen.se/en/) and Statistics Sweden (https://www.scb.se/en/), which are government agencies providing access to the linked healthcare registers. The data will be shared on reasonable request in an application made to any of the steering groups of the NPCR and PCBase (contact npcr@npcr.se or par.stattin@uu.se). To request data or analytic code from this study, contact the corresponding author. For detailed information, please see www.npcr.se/in-english, where registration forms, manuals, and annual reports from NPCR are available alongside a full list of publications from PCBase.
